# Histological diagnostic criterion for chronic endometritis based on the clinical outcome

**DOI:** 10.1186/s12905-021-01239-y

**Published:** 2021-03-04

**Authors:** Kimiko Hirata, Fuminori Kimura, Akiko Nakamura, Jun Kitazawa, Aina Morimune, Tetsuro Hanada, Akie Takebayashi, Akiko Takashima, Tsukuru Amano, Shunichiro Tsuji, Shoji Kaku, Ryoji Kushima, Takashi Murakami

**Affiliations:** 1 Department of Obstetrics and Gynaecology, Shiga University of Medical Science, Seta Tsukinowa-Cho, Otsu, Shifga 520-2192 Japan; 2 Goto Ladies Clinic, 4-13 Hakubaicho, Takatsuki, Osaka 569-1116 Japan; 3 Department of Obstetrics and Gynaecology, National Hospital Organization Shiga Hospital, 255 Gochi-cho, Higashioumi, Shiga 527-8505 Japan; 4 Department of Clinical Laboratory Medicine and Division of Diagnostic Pathology, Shiga University of Medical Science, Seta Tsukinowa-Cho, Otsu, Shiga 520-2192 Japan

**Keywords:** Chronic endometritis, Diagnostic criterion, Infertility, Plasma cell

## Abstract

**Background:**

The diagnostic criteria of chronic endometritis remain controversial in the treatment for infertile patients.

**Methods:**

A prospective observational study was conducted in a single university from June 2014 to September 2017. Patients who underwent single frozen-thawed blastocyst transfer with a hormone replacement cycle after histological examination for the presence of chronic endometritis were enrolled. Four criteria were used to define chronic endometritis according to the number of plasma cells in the same group of patients: 1 or more (≥ 1) plasma cells, 2 or more (≥ 2), 3 or more (≥ 3), or 5 or more (≥ 5) in 10 high-power fields. Pregnancy rates, live birth rates, and miscarriage rates of the non-chronic endometritis and the chronic endometritis groups defined with each criterion were calculated. A logistic regression analysis was performed for live births using eight explanatory variables (seven infertility factors and chronic endometritis). A receiver operating characteristic curve was drawn and the optimal cut-off value was calculated.

**Results:**

A total of 69 patients were registered and 53 patients were finally analyzed after exclusion. When the diagnostic criterion was designated as the presence of ≥ 1 plasma cell in the endometrial stroma per 10 high-power fields, the pregnancy rate, live birth rate, and miscarriage rate were 63.0% vs. 30.8%, 51.9% vs. 7.7%, and 17.7% vs. 75% in the non-chronic and chronic endometritis groups, respectively. This criterion resulted in the highest pregnancy and live birth rates among the non-chronic endometritis and the smallest *P* values for the pregnancy rates, live birth rates, and miscarriage rates between the non-chronic and chronic endometritis groups. In the logistic regression analysis, chronic endometritis was an explanatory variable negatively affecting the objective variable of live birth only when chronic endometritis was diagnosed with ≥ 1 or ≥ 2 plasma cells per 10 high-power fields. The optimal cut-off value was obtained when one or more plasma cells were found in 10 high-power fields (sensitivity 87.5%, specificity 64.9%).

**Conclusions:**

Chronic endometritis should be diagnosed as the presence of ≥ 1 plasma cells in 10 high-power fields. According to this diagnostic criterion, chronic endometritis adversely affected the pregnancy rate and the live birth rate.

## Background

Chronic endometritis (CE) is defined as slight inflammation of the endometrium and is generally agreed that the presence of plasma cells within the endometrial stroma is the most useful histologic criterion for diagnosis [1–7]. Although patients with CE have no or subtle clinical symptoms, and no clinical significance has yet been found, there have recently been many reports that show its relationship with infertility and implantation failure [4, 8–16].

Epidemiologically, CE is recognized in 2.8% to 67.6%, from a very low frequency to a very high frequency, of patients with infertility and implantation failure [4, 8, 11–16]. Bacterial infection is related to the cause of CE, because many cases of CE are cured by antibiotics [8–10, 15, 17–19]. It has been reported that the clinical outcomes of in vitro fertilized embryo transfer are improved when cure of CE is confirmed after administration of antibiotics for recurrent implantation failure (RIF) [10, 15, 17–19]. However, on the other hand, there have been reports that administration of antibiotics is ineffective, and reports that CE does not affect fertility at all [4, 8]. Although the cause of the differences in the research results regarding the effects of CE on fertility may be due to different patient backgrounds and subjects in each study, we considered that the biggest problem is the difference in the diagnostic criteria for CE in each study. In many previous clinical studies of CE, the study subjects were patients with RIF, and the clinical outcomes were compared between the group cured with antibiotics and the persistent group [8–10, 13, 17]. However, antibiotics cannot be administered without establishing diagnostic criteria in advance. Thus, this methodology interferes with the determination of CE criteria depending on the clinical outcomes. Moreover, it is also difficult to purely evaluate the effect of CE on implantation when the control group is defined as patients with RIF without CE, since the pregnancy rate in patients with RIF will be lower in subsequent treatment cycles due to the presence of causes other than CE for implantation failure. For these reasons, there appear to be no uniform criteria based on clinical outcomes that are accepted worldwide.

Therefore, the present study examined whether or not the pregnancy rate, live birth rate and miscarriage rate differed between non-CE and CE when different diagnostic criteria were set for the number of plasma cells in the endometrial stroma and the aim of the present study was to establish the most appropriate diagnostic criteria for CE.

## Methods

This study was approved by the Ethics Committee of Shiga University of Medical Science (approved number 2014-090). All clinical studies were conducted according to the Declaration of Helsinki for Medical Research involving Human Subjects. Informed written consent was obtained from the participants. The patients were enrolled from June 2014 to September 2017.

**Fig. 1:**
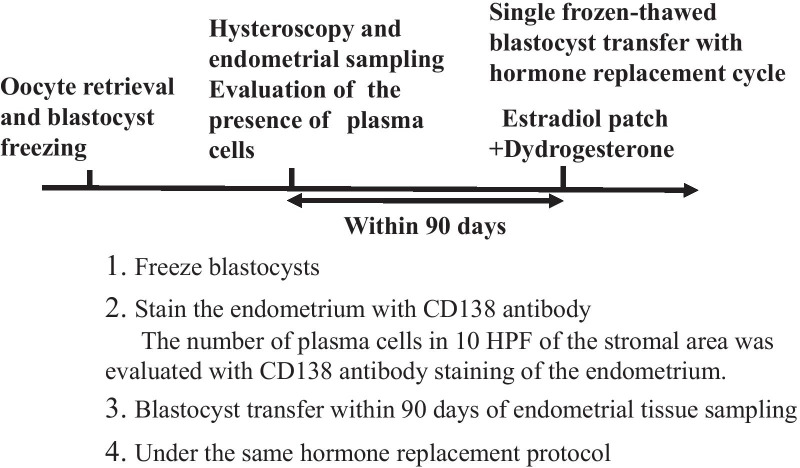
Protocol for the present study. Blastocysts were frozen following oocyte retrieval. Hysteroscopy and endometrial sampling were performed, and the number of plasma cells in 10 high-power fields (HPFs) of stromal area was evaluated with CD138 antibody staining of the endometrium. Single frozen-thawed blastocyst transfer was performed within 90 days after the endometrial evaluation with a hormone replacement cycle

As shown in Fig. 1, IVF or intracytoplasmic sperm injection was performed with a gonadotropin releasing hormone (GnRH) agonist protocol or a GnRH antagonist protocol, and the blastocysts were frozen.

The subjects were patients under 41 years of age who agreed and underwent the in vitro fertilization-embryo transfer (IVF-ET) protocol of our department, which includes routine hysteroscopy and local endometrial curettage (injury) before first frozen thawed embryo transfer.

**Fig. 2:**
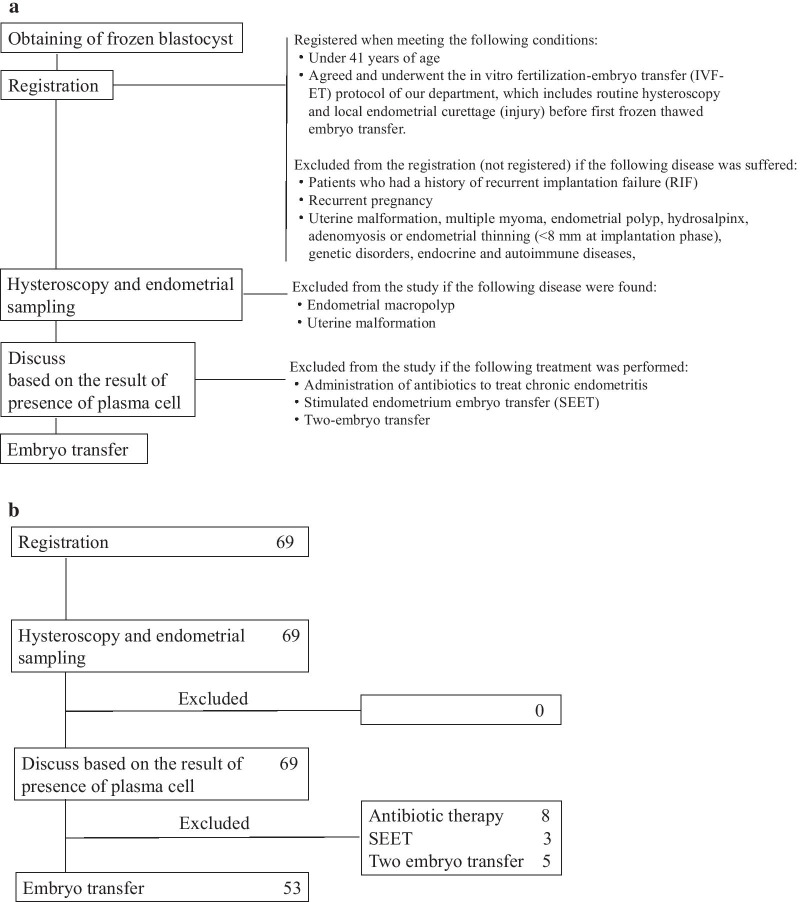
**a** Flow of patient registration and exclusion. The subjects were patients under 41 years of age who agreed and underwent the in vitro fertilization-embryo transfer (IVF-ET) protocol of our department, which includes routine hysteroscopy and local endometrial curettage (injury) before first frozen thawed embryo transfer. Patients who had a history of RIF, recurrent pregnancy loss (RPL) or diseases suspected to cause implantation failure such as submucosal myoma, adenomyosis, uterine malformation, or endometrial thinning (< 8 mm at implantation phase) were not enrolled. Genetic disorders, endocrine diseases and autoimmune diseases were not enrolled either. The presence of endometrial macropolyps and uterine malformations were evaluated by hysteroscopy, and patients with these diseases were excluded from this study at that moment. Before blastocyst transfer, patients were discussed with doctors again with respect to the treatment options, based on the result of existence of plasma cell in the stroma of the endometrium, and when the patient wanted to add the treatment which might modify the endometrial receptivity such as administration of antibiotics, or stimulated endometrium embryo transfer, the patients were excluded from the study. The patients who want to treat two-embryo transfer were also excluded to limit the study of treatment outcomes for single blastocyst transfer. **b** The numbers of patients registered and excluded. Sixty-nine patients were recruited, and 16 patients were excluded. Fifty-seven patients were finally analyzed

The criteria for registration were set accordingly to a previous report with some modification [20]. Briefly, patients who had a history of RIF, recurrent pregnancy loss (RPL) or diseases suspected to cause implantation failure such as uterine malformation, multiple myoma, endometrial polyp, hydrosalpinx, adenomyosis with over a uterine wall > 2.5 cm thick or endometrial thinning (< 8 mm at implantation phase) detected by ultrasonography [21–26], were not enrolled. Genetic disorders, endocrine diseases and autoimmune diseases were not enrolled either [27, 28] (Fig. 2a). RIF was defined as the failure of clinical pregnancy after 4 good quality embryo transfers, with at least three fresh or frozen IVF cycles, as per Coughlan et al. [29]. RPL was defined as the patient with 3 or more miscarriage [30].

After the identification of the ovulation day as reported previously [20], hysteroscopy and endometrial tissue collection were performed 5–9 days after ovulation. Whether endometrial macropolyps and uterine malformations were present was determined by hysteroscopy, and patients with these diseases were excluded from this study at that moment [23, 24] (Fig. 2a). Immediately after hysteroscopy, the tissue around the center of the anterior endometrium was collected with 4.5 J. A. M. W Type Uterine Curettes, and immunostaining of this sample for CD138 was performed, as reported previously [1, 20, 31, 32]. One pathologist examined this tissue to determine how many plasma cells there were in 10 random HPFs (Olympus BX-41; hpf diameter = 0.55 mm; hpf area = 0.24 mm^2^) of the endometrial stromal area.

Before blastocyst transfer, patients again discussed their treatment options with doctors based on the findings concerning the presence of plasma cells in the stroma of the endometrium, and when the patient wanted to add treatment that might modify the endometrial receptivity, such as the administration of antibiotics [10, 13] or stimulated endometrium embryo transfer (SEET) [33], the patients were excluded from the study (Fig. 2a). Patients who wished to undergo two-embryo transfer were also excluded in order to limit the study’s treatment outcomes to single blastocyst transfer (Fig. 2a).

**Fig. 3:**
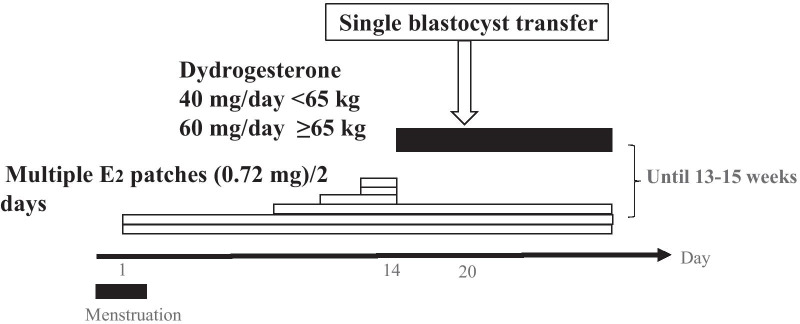
Protocol of single frozen-thawed blastocyst transfer with a hormone replacement cycle. Multiple estradiol patches are started on days 2–3 of menstruation and increased gradually. When the endometrial thickness reaches 8 or more mm, oral administration of dydrogesterone (DYD) is started. Single blastocyst transfer is done 6 days after DYD is started

Blastocysts were transferred within 90 days of endometrial tissue collection. An estradiol patch was started on days 2–3 of menstruation and increased gradually (Fig. 3). When the endometrial thickness reached 8 or more mm, oral administration of dydrorgesterone (DYD) was started (40 mg/day for patients weighing less than 65 kg or 60 mg for those weighing 65 kg or more). Single frozen-thawed blastocyst transfer was performed six days after DYD administration, and a blood human chorionic gonadotropin (hCG) test was performed two weeks after blastocyst transfer. When hCG was detected, transvaginal ultrasonography was performed within one week from that day, and the presence of a gestational sac in utero was considered to indicate pregnancy. When pregnancy was recognized, administration of these hormones was continued until 13 to 15 weeks of pregnancy. When the patients did not conceive or miscarriage resulted, their administration was discontinued as appropriate.

After obtaining the clinical outcomes of blastocyst transfer, the effect of the presence of plasma cell on the outcome was evaluated. We set four different diagnostic criteria for the number of plasma cells existing in the stromal area per 10 HPFs and determined which criteria matched the clinical data best. Diagnostic criteria were decided with reference to past reports. Four different criteria for CE were used, according to the number of plasma cells: the first diagnostic criterion called for diagnosing CE when ≥ 1 plasma cell was found among 10 HPFs and non-CE when no plasma cells were found [34]; the second diagnostic criterion called for diagnosing CE when ≥ 2 plasma cells were found among 10 HPFs and non-CE when ≤ 1 plasma cell was found [35]; the third diagnostic criterion called for diagnosing CE when ≥ 3 plasma cells were found among 10 HPFs and non-CE when ≤ 2 plasma cells were found [35]; and the fourth diagnostic criterion called for diagnosing CE when ≥ 5 plasma cells were found among 10 HPFs and non-CE when ≤ 4 plasma cells were found [11]. Pregnancy rates, live birth rates, and miscarriage rates were calculated in the non-CE and CE groups for each criterion and outcomes were compared among four criteria.

A multivariate logistic regression analysis was performed for eight explanatory variables, which included seven infertility factors (male factor, tubal factor, endometriosis, ovarian factor, anti-sperm antibody-positive, fertilization failure, unexplained infertility) and CE, with respect to the objective variable of live birth to obtain factors that worked negatively for each criterion, as previously reported [20].

In addition, the sensitivity and specificity of the non-CE group for live birth were calculated for each of these diagnostic criteria, and a receiver operating characteristic (ROC) curve was drawn from them. Based on the results, the area under the curve (AUC) was calculated, and the optimal cut-off values were determined.

### Statistically analyses

The target number of participants in the present study was calculated based on a retrospective study reported by Cicinelli et al. [10]. According to their report, when CE was cured with antibiotics, the ongoing pregnancy rate was 60.8% (28/46), but when it persisted, the rate was 13.3% (2/15); there was a significant difference between them. Based on these results, the number of patients required for enrollment was calculated using software provided by the Department of Biostatistics, Vanderbilt University (http://biostat.mc.vanderbilt.edu/wiki/Main/PowerSampleSize). In the section of Dichotomous, independent, prospective, two proportion, and Fisher’s exact test were selected to measure the sample size. An *α* of 0.05 was selected as the probability of falsely rejecting the null hypothesis, with 0.8 for *power* (the probability of always rejecting the null hypothesis if the null hypothesis is false in the statistical hypothesis test), 0.605 for *P0* (the probability of the outcome for a control patient in prospective studies), and 0.133 for *P1* (the probability of the outcome in an experimental subject in prospective studies). When a value of 1 was selected for *m* (the ratio of control to experimental subjects for independent prospective studies), it was calculated that enrollment of 19 cases was necessary for each group. When m was chosen as 0.56, 0.36, 0.26 and 0.55, the number of cases required for control (non-CE) and CE became 26 and 15, 33 and 12, 42 and 11, and 26 and 15, respectively.

Statistical analysis was performed using Graph Pad Prism 5 (GraphPad Software Inc., La Jolla, CA). The normality of the distribution of each dataset was analyzed using the Kolmogorov–Smirnov test, and then Student’s *t*-test or the non-parametric Mann–Whitney U test was used depending on the distribution pattern. The significance of differences in pregnancy, live birth, and abortion rates between the non-CE and CE groups was examined using Fisher’s test. A significant difference was defined as a *P* value less than 0.05.

The SSPS statistics software program, version 25 (IBM, Chicago, IL, USA) was used for the multivariate logistic regression analysis. Odds ratios and *P* values were calculated. A significant difference was considered present at a *P* value < 0.05.

The JMP® software program (SAS Institute Inc. Cary, NC, USA) was used to depict the ROC curve.

## Results

A total of 69 patients were registered (Fig. 2b). Of these, patients who wanted treatment with antibiotics, SEET, or two-embryo transfer, were excluded, and 53 patients were finally analyzed.

When CE was defined as ≥ 1 plasma cells in 10 HPFs, there were 27 non-CE patients and 26 CE patients. When CE was defined as ≥ 2 plasma cells in 10 HPFs, ≥ 3 plasma cells in 10 HPFs, or ≥ 5 plasma cells in 10 HPFs, the numbers of non-CE patients and CE patients were 34 and 19, 39 and 14, and 42 and 11, respectively. These met the statistically required numbers**.**

When the diagnostic criterion for CE was the presence of ≥ 1 plasma cells in 10 HPFs, there were no differences between the two groups in patients’ background except parity (Table 1). The pregnancy rate, live birth rate, and miscarriage rate were 63.0% vs 30.8% (*P* = 0.028), 51.9% vs 7.7% (*P* = 0.0007), and 17.7% vs 75% (*P* = 0.0099) in the non-CE and CE groups, respectively. The pregnancy rate and live birth rate were significantly lower in the CE group, and the miscarriage rate was significantly higher in the CE group.

**Table 1:** Patients’ characteristics and clinical outcomes when CE was defined as ≥ 1 PCs in 10 HPFs

	Non-CE	CE	*P* value
	N = 27	N = 26	
Age (y), Mean ± SD	35.0 ± 3.30	36.2 ± 3.45	0.2
Gravidity, Median, IQR	0 (0–2)	0 (0–1)	0.23
Parity, Median, IQR	0 (0–0)	0 (0–1)	0.039
BMI (kg/m^2^), Median, IQR	21.4 (18.9–23.7)	21.8 (20.2–26.6)	0.24
Smoking habit (%)	0	0	1.00
FSH (mIU/mL), Median, IQR	8.1 (4.8–24)	9.1 (5–12.5)	0.2
Previous OPU cycles, Median IQR	1 (1–4)	1.5 (1–2.25)	0.72
EM thickness (mm), Mean ± SD	10.3 ± 1.60	10.5 ± 2.38	0.75
Rate of good blastocysts (%)	59.3 (16/27)	65.4 (17/26)	0.78
Infertility cause			
Tubal factor	5	10	0.14
Ovarian factor	4	4	1
Endometriosis	7	6	1
ASA	0	0	1
Fertilization failure	1	1	1
Male factor	7	6	1
Unexplained fertility	10	4	0.12
Pregnancy rate (%)	63.0 (17/27)	30.8 (8/26)	0.028
Live birth rate (%)	51.9 (14/27)	7.7 (2/26)	0.0007
Miscarriage rate (%)	17.7 (3/17)	75 (6/8)	0.0099

*CE* chronic endometritis, *PCs* plasma cells, *non-CE* non chronic endometritis, *HPF* high-power field, *SD* standard deviation, *IQR* interquartile range, *BMI* body mass index, *FSH* follicle stimulate hormone, *OPU* ova pick up, *EM* endometrial, *ASA* anti-sperm antibody positive

When the diagnostic criterion for CE was the presence of ≥ 2 plasma cells in 10 HPFs, there were no differences between the two groups in patients’ background except age, parity, and the rate of tubal factor as infertility cause (Table 2). The pregnancy rate, live birth rate, and miscarriage rate were 58.8% vs 26.3% (*P* = 0.043), 44.1% vs 5.3% (*P* = 0.0041), and 25% vs 80% (*P* = 0.04) in the non-CE and CE groups, respectively. The pregnancy rate and live birth rate were significantly lower in the CE group, and the miscarriage rate was significantly higher in the CE group.

**Table 2:** Patients’ characteristics and clinical outcomes when CE was defined as ≥ 2 PCs in 10 HPFs

	Non-CE	CE	*P* value
	N = 34	N = 19	
Age (y), Median, IQR	36 (32.5–37)	38 (35–39)	0.028
Gravidity, Median, IQR	0 (0–0.25)	0 (0–1)	0.13
Parity, Median, IQR	0 (0–1)	0 (0–1)	0.0023
BMI (kg/m^2^), Median, IQR	21.8 (19.6–24.1)	21.0 (20.1–25.9)	0.83
Smoking habit (%)	0	0	1
FSH (mIU/mL), Median, IQR	8.85 (7.4–9.7)	8.8 (7.4–10.3)	0.97
Previous OPU cycles, Median IQR	1.5 (1–4)	1 (1–2)	0.33
EM thickness (mm), Mean ± SD	10.5 ± 2.05	10.2 ± 1.95	0.55
Rate of good blastocysts (%)	61.2 (21/34)	63.1 (12/19)	0.99
Infertility cause			
Tubal factor	6	9	0.029
Ovarian factor	5	3	1
Endometriosis	8	5	1
ASA	0	0	1
Fertilization failure	1	1	1
Male factor	11	2	0.10
Unexplained fertility	11	3	0.33
Pregnancy rate (%)	58.8 (20/34)	26.3 (5/19)	0.043
Live birth rate (%)	44.1 (15/34)	5.3 (1/19)	0.0041
Miscarriage rate (%)	25 (5/20)	80 (4/5)	0.04

*CE* chronic endometritis, *PCs* plasma cells, *non-CE* non chronic endometritis, *HPF* high-power field, *SD* standard deviation, *IQR* interquartile range, *BMI* body mass index, *FSH* follicle stimulate hormone, *OPU* ova pick up, *EM* endometrial, *ASA* anti-sperm antibody positive

When the diagnostic criterion for CE was the presence of ≥ 3 plasma cells in 10 HPFs, there were no differences between the two groups in patients’ background except age, gravidity, and parity (Table 3). The pregnancy rate, live birth rate, and miscarriage rate were 53.9% vs 28.6% (*P* = 0.13), 38.5% vs 7.1% (*P* = 0.041), and 28.6% vs 75% (*P* = 0.12) in the non-CE and CE groups, respectively. The live birth rate was significantly lower in the CE group, but there were no differences in the pregnancy rate and the miscarriage rate between the groups.

**Table 3:** Patients’ characteristics and clinical outcomes when CE was defined as ≥ 3 PCs in 10 HPFs

	Non-CE	CE	*P* value
	N = 39	N = 14	
Age (y), Median, IQR	36 (33–37)	38 (37–39)	0.0051
Gravidity, Median, IQR	0 (0–0)	0.5 (0–2)	0.04
Parity, Median, IQR	0 (0–0)	0.5 (0–1)	0.0006
BMI (kg/m^2^), Median, IQR	21.9 (19.6–24.5)	20.9 (19.9–24.1)	0.54
Smoking habit (%)	0	0	1
FSH (mIU/mL), Median, IQR	8.6 (6.7–9.7)	9.05 (7.83–10.4)	0.18
Previous OPU cycles, Median IQR	1 (1–4)	1.5 (1–3.3)	0.93
EM thickness (mm), Mean ± SD	10.5 ± 2.0	10.0 ± 2.0	0.39
Rate of good blastocysts (%)	64.1 (25/39)	57.1 (8/14)	0.75
Infertility cause			
Tubal factor	10	5	0.50
Ovarian factor	6	2	1
Endometriosis	9	4	0.73
ASA	0	0	1
Fertilization failure	1	1	0.4
Male factor	11	2	0.47
Unexplained fertility	11	3	0.74
Pregnancy rate (%)	53.9 (21/39)	28.6 (4/14)	0.13
Live birth rate (%)	38.5 (15/39)	7.1 (1/14)	0.041
Miscarriage rate (%)	28.6 (6/21)	75 (3/4)	0.12

*CE* chronic endometritis, *PCs* plasma cells, *non-CE* non chronic endometritis, *HPF* high-power field, *SD* standard deviation, *IQR* interquartile range, *BMI* body mass index, *FSH* follicle stimulate hormone, *OPU* ova pick up, *EM* endometrial, *ASA* anti-sperm antibody positive

When the diagnostic criterion for CE was the presence of ≥ 5 plasma cells in 10 HPFs, there were no differences between the two groups in patients’ background except age and parity (Table 4). The pregnancy rate, live birth rate, and miscarriage rate were 50% vs 36.4% (*P* = 0.51), 35.7% vs 9.1% (*P* = 0.14), and 28.6% vs 75% (*P* = 0.12) in the non-CE and CE groups, respectively. There were no significant differences in the pregnancy rate, live birth rate, and miscarriage rate.

**Table 4:** Patients’ characteristics and clinical outcomes when CE was defined as ≥ 5 PCs in 10 HPFs

	Non-CE	CE	*P* value
	N = 42	N = 11	
Age (y), Median, IQR	36 (33–37)	38 (37–39)	0.04
Gravidity, Median, IQR	0 (0–2)	0 (0–2)	0.15
Parity, Median, IQR	0 (0–1)	0 (0–2)	0.023
BMI (kg/m^2^), Median, IQR	21.8 (19.6–24.1)	20.9 (20.1–25.9)	0.90
Smoking habit (%)	0	0	1
FSH (mIU/mL), Median, IQR	8.7 (6.9–9.8)	9 (7.6–10.3)	0.42
Previous OPU cycles, Median IQR	1.5 (1–4)	1 (1–3)	0.46
EM thickness (mm), Mean ± SD	10.4 ± 2.0	10.2 ± 2.1	0.77
Rate of good blastocysts (%)	59.5 (25/42)	72.3 (8/11)	0.5
Infertility cause			
Tubal factor	11	4	0.71
Ovarian factor	6	2	0.67
Endometriosis	11	2	0.71
ASA	0	0	1
Fertilization failure	2	0	1
Male factor	12	1	0.26
Unexplained fertility	11	3	1
Pregnancy rate (%)	50 (15/42)	36.4 (4/11)	0.51
Live birth rate (%)	35.7 (15/42)	9.1 (1/11)	0.14
Miscarriage rate (%)	28.6 (6/21)	75 (3/4)	0.12

*CE* chronic endometritis, *PCs* plasma cells, *non-CE* non chronic endometritis, *HPF* high-power field, *SD* standard deviation, *IQR* interquartile range, *BMI* body mass index, *FSH* follicle stimulate hormone, *OPU* ova pick up, *EM* endometrial, *ASA* anti-sperm antibody positive

According to the multivariate logistic regression analysis, CE was the only explanatory variable that negatively affected the objective variables of live birth when CE was diagnosed with the diagnostic criteria of ≥ 1 or ≥ 2 plasma cells per 10 HPFs, although no independent variable was detected as a negative factor when CE was diagnosed using other criteria. The odds ratio (*P* value, [95% confidence interval]) for live birth was 0.081 (*P* = 0.016, [0.01–0.625]) when CE was diagnosed with ≥ 1 plasma cell per 10 HPFs and 0.064 (*P* = 0.044, [0.004–0.93) when CE was diagnosed with ≥ 2 plasma cells per 10 HPFs (Table 5).

**Table 5:** A multivariate logistic regression analysis for variables negatively affecting live birth in each criterion

Diagnostic criterion	Variable	Odds ratio	95% CI	*P* value
≥ 1 plasma cells in 10 HPFs	CE	0.081	0.010–0.625	0.016
≥ 2 plasma cells in 10 HPFs	CE	0.064	0.004–0.928	0.044

*CE* chronic endometritis, *CI* confidential interval, *HPF* high power field

**Fig. 4:**
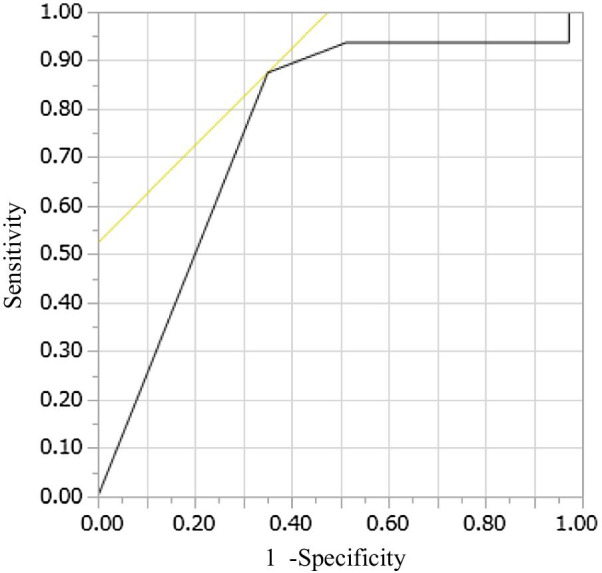
A receiver operating characteristic curve of the non-CE group for live birth. The sensitivity and specificity of the non-CE group for live birth were calculated for each of these diagnostic criteria, and a receiver operating characteristic (ROC) curve was drawn. The area under the curve of the ROC curve was 0.785. The optimal cut-off value was obtained when ≥ 1 plasma cell was found in 10 HPFs (sensitivity of 87.5% and specificity of 64.9%)

The AUC of the ROC curve was 0.785. The optimal cut-off value was obtained when ≥ 1 plasma cell was found among 10 HPFs (sensitivity of 87.5% and specificity of 64.9%) (Fig. 4).

## Discussion

In the present study, diagnostic criteria for CE using endometrial specimens were prospectively evaluated based on clinical outcomes in patients undergoing a uniform program of single blastocyst transfer in hormone replacement cycles after excluding participants with RIF, RPL, and diseases suspected to cause implantation failure when participants were enrolled. The pregnancy rate and live birth rate of the non-CE group were highest when the diagnostic criterion for CE was the presence of ≥ 1 plasma cells in 10 HPFs. The pregnancy rate and live birth rate of the non-CE group decreased gradually as the minimum number of plasma cells in 10 HPFs for the diagnostic criterion for CE increased from ≥ 2 to ≥ 5. On the other hand, the miscarriage rate of the non-CE group was the lowest when the diagnostic criterion for CE was the presence of ≥ 1 plasma cells in 10 HPFs. All *P* values for the pregnancy rate, live birth rate, and miscarriage rate were the smallest when the diagnostic criterion of CE was the presence of ≥ 1 plasma cells in 10 HPFs. Their *P* values increased gradually as the minimum number of plasma cells in 10 HPFs for the diagnostic criterion for CE increased from ≥ 1 to ≥ 5. When the diagnostic criterion for CE was the presence of ≥ 5 plasma cells in 10 HPFs, there were no significant differences in the pregnancy rates and live birth rates.

In the multivariate logistic regression analysis, CE was an explanatory variable negatively affecting the objective variable of live birth only when CE was diagnosed based on the presence of ≥ 1 or ≥ 2 plasma cells per 10 HPFs. In addition, in the evaluation of the ROC curve, the optimal cut-off was determined to be ≥ 1 plasma cell per 10 HPFs. Taken together, these findings suggest that CE should be diagnosed when ≥ 1 plasma cell is found among 10 HPFs, and CE had a detrimental effect on the clinical outcomes of in vitro fertilization when it was diagnosed with this criterion.

In the present study, the live birth rate and miscarriage rate did differ among the three groups although there was no statistically difference in the pregnancy rate (Table 6). The live birth rates were higher in the patients with no plasma cells than in those with 1–4 or ≥ 5 plasma cells per 10 HPFs when compared between the two groups although there were no marked differences between the patients with 1–4 and ≥ 5 plasma cells per 10 HPFs with respect to these rates. The pregnancy rate and miscarriage rate did not differ between any combination of the 3 groups (Table 6). This indicates that when plasma cells were detected, the live birth rate decreased in the same way, regardless of the number of plasma cells detected.

**Table 6:** Clinical outcomes among three groups of the patients with 0, 1–4, and ≥ 5 plasma cells

Number of plasma cells	0	1–4	≥ 5	*P* value
	N = 27	N = 15	N = 11	
Pregnancy rate (%)	63.0 (17/27)	33.3 (4/15)	36.4 (4/11)	0.057
Live birth rate (%)	51.9 (14/27)*,**	8.3 (1/15)	9.1 (1/11)	0.0022
Miscarriage rate (%)	17.6 (3/14)	75 (3/4)	75 (3/4)	0.049

*P < 0.05, Statistically significant difference was detected when comparing between the group of the patients with 0 plasma cell and the group of the patients with ≥ 5 plasma cells

**P < 0.01, Statistically significant difference was detected when comparing between the group of the patients with 0 plasma cell and the group of the patients with 1–4 plasma cells

In studies based on the detection of plasma cells, the criteria were ≥ 1 plasma cell per section [10, 12], ≥ 1 plasma cell in the stromal area among 10 HPFs [6], ≥ 5 plasma cells in the stromal area among 20 HPFs [34], and ≥ 5 plasma cells in the stromal area among 10 HPFs [11], showing marked variety among studies. In these reports, the efficacy of antibiotics on the clinical outcomes or the prevalence of CE was determined. However, in those studies, antibiotics could not be administered without establishing diagnostic criteria in advance, and the prevalence of CE could not be determined either without establishing diagnostic criteria in advance. Thus, this methodology interferes with the determination of CE criteria based on the clinical outcomes. Recently, a few retrospective studies have established diagnostic criteria based on the clinical outcomes without the usage of antibiotics, even when CD138-positive cells were found in the endometrial stroma [36, 37]. In one study, CD138-positive cells were counted, excluding positive cells in the lumen and glands, in patients with RIF. The results were expressed as the number of CD138 cells per section. Based on the ROC analysis, the optimal cut-off value was concluded to be 0.5 (sensitivity of 77.2% and specificity of 52.5%) [37]. This result was similar to our own in that CE was diagnosed when even 1 plasma cell was found.

Another study determined the diagnostic criterion in patients undergoing in vitro fertilization, including RIF. In that study, 30 HPFs were evaluated for each sample, resulting in a criterion of ≥ 5 CD138-positive cells in ≥ 1 HPF [36]. This study asserted the importance of finding the accumulation of five or more plasma cells in a given area. This analysis was conducted based on a different point of view than our study.

However, it might be difficult to evaluate the effect of CE on implantation in the above-mentioned studies when control group includes RIF, since the pregnancy rate in patients with RIF will decrease in subsequent treatment cycles due to the presence of implantation failure causes other than CE.

The present study included patients undergoing IVF, but excluded patients with RIF, RPL, and causes of implantation failure when enrolled. This is the decisive difference between the present study and the previous studies. As a result, for such patients, we have proven that those with even one plasma cell in the endometrial stromal area should be diagnosed with CE because of their lower pregnancy rate and live birth rate. This result also indicates that the presence of CE adversely affects implantation, regardless of the presence of RIF, in the patients treated with IVF.

This study was a prospective study that excluded patients who were likely to affect implantation at the registration stage. A single blastocyst is transferred within three months after the diagnosis of CE in a unified hormone replacement cycle. Furthermore, in the present study, three different analyses—the evaluation of differences in clinical outcomes among the four criteria, a logistic regression analysis for the live birth rate and the determination of the optimal cut-off value using the ROC curve—indicated the same diagnostic criteria for the diagnosis of CE. These are the strengths of the present study.

However, several limitations associated with the present study also warrant mention. First, the number of participants were relatively small compared with other studies, although the number of samples required for this prospective study was calculated based on the results of a similar retrospective clinical study conducted previously.

Second, this study analyzed the clinical outcomes in the hormone replacement cycle using DYD. As a recent report suggested, the efficacy of the oral administration of DYD as luteal support in fresh embryo transfer is equivalent or superior to that of a progesterone vaginal suppository [38–40]. However, the results of the present study may be limited by this hormone replacement cycle.

Third, due to ethical issues, the results of CE were disclosed to patients, and when desired, treatment was performed, such as the administration of antibiotics. Patients who wished to be treated with antibiotics might have been potentially intractable cases, so the exclusion of these patients may have resulted in bias.

Now that the diagnostic criterion has been established, our next research topic will be to determine whether or not the clinical outcomes of patients diagnosed with CE based on the diagnostic criterion are improved after these patients are cured, such as by treatment with antibiotics. It will be particularly necessary to confirm whether or not the pregnancy continues to term following the successful treatment of CE.

Finally, various immunocompetent cells are present in the endometrium and are involved in the establishment of pregnancy [41–44]. CE causes the abnormal distribution of immunocompetent cells in the endometrium of implantation phase [45, 46]. Research is currently underway using the term “endometritis”, which is primarily caused by bacteria. In this sense, “impaired inflammatory state of the endometrium (IISE)” might be a more appropriate term than “endometritis”, as it directly indicates “inflammation of the endometrium”, regardless of the cause [42, 43]. We may need to revise concepts, including terminology, with respect to research being conducted on inflammation in the uterus and the evaluation of its effect on fertility. We intend to explore this issue in a future study.

## Conclusions

The present prospective study showed that the pregnancy rate, live birth rate and miscarriage rate of the non-CE group differed depending on the diagnostic criteria used for CE. Based on our evaluation of the differences in the pregnancy rate, live birth rate and miscarriage rate among the four criteria as well as the results of a logistic regression analysis for live birth and the optimal cut-off value analyzed based on the ROC curve, CE should be diagnosed in the presence of ≥ 1 plasma cell per 10 HPFs. With this diagnostic criterion, it was shown that CE adversely affected the pregnancy rate and live birth rate in IVF, even in patients without evidence of RIF, RPL or diseases suspected of causing implantation failure.

## Data Availability

We can provide the raw data. The datasets used and/or analyzed during the current study available from the corresponding author on reasonable request.

## Abbreviations

CE: Chronic endometritis
DYD: Dydrorgesterone
HPF: High-power field
IVF: In vitro fertilization
RIF: Recurrent implantation failure
RPL: Recurrent pregnancy loss
